# Clinicians' management strategies for patients with dyspepsia: a qualitative approach

**DOI:** 10.1186/1471-230X-5-15

**Published:** 2005-05-15

**Authors:** Kerstin Knutsson, Bodil Ohlsson, Margareta Troein

**Affiliations:** 1 Faculty of Odontology, Centre for Oral Health Sciences, Malmö University, Malmö, Sweden; 2 Department of Clinical Sciences, Gastroenterology and Hepatology Division, Malmö University Hospital, Lund University, Lund, Sweden; 3 Department of Community Medicine, Malmö University Hospital, Lund University, Lund, Sweden

**Keywords:** gastrointestinal tract, decision-making, dyspepsia, qualitative evaluation

## Abstract

**Background:**

Symptoms from the upper gastrointestinal tract are frequently encountered in clinical practice and may be of either organic or functional origin. For some of these conditions, according to the literature, certain management strategies can be recommended. For other conditions, the evidence is more ambiguous. The hypothesis that guided our study design was twofold: Management strategies and treatments suggested by different clinicians vary considerably, even when optimal treatment is clear-cut, as documented by evidence in the literature. Clinicians believe that the management strategies of their colleagues are similar to their own.

**Methods:**

Simulated case histories of four patients with symptoms from the upper gastrointestinal tract were presented to 27 Swedish clinicians who were specialists in medical gastroenterology, surgery, and general practice and worked at three hospitals in the southern part of Sweden. The patients' histories contained information on the patient's sex and age and the localisation of the symptoms, but descriptions of subjective symptoms and findings from examinations differed from history to history. Interviews containing open-ended questions were conducted.

**Results:**

For the same patient, the management strategies and treatments suggested by the clinicians varied widely, as did the strategies suggested by clinicians in the same speciality. Variation was more pronounced if the case history noted symptoms but no organic findings than if the case history noted unambiguous findings and symptoms. However, even in cases with a consensus in the scientific literature on treatment, the variations in clinicians' opinion on management were pronounced.

**Conclusion:**

Despite these variations, the clinicians believed that the decisions made by their colleagues would be similar to their own. The overall results of this study indicate that we as researchers must make scientific evidence comprehensible and communicate evidence so that clinicians are able to interpret and implement it in practice. Of particular significance is that scientific evidence leads to an evidence-based care which is effective clinical practice and to the promotion of health from the perspective of the patient, together with cost-effectiveness as a priority.

## Background

The quality of health care is determined by two main factors: the quality of the judgements and decisions that determine what actions are to be taken, and the quality with which those actions are executed [1]. Within health care, there are wide variations in clinicians' judgements on diagnosis and in the management of patients with the same symptoms and diagnosis [2]. These variations are seen within different disciplines, among both experts and novices. In spite of this evidence, clinicians generally believe that the decisions made by their colleagues would be similar to their own, and hence they assume that there is a broad consensus in medical practice [2].

Patients with symptoms from the upper gastrointestinal tract are regularly seen in clinical practice. The symptoms may be either of organic origin – e.g. ulcer disease, oesophagitis, and malignancies in the oesophagus and the ventricle – or of functional origin [3]. Dyspepsia is a collective term and includes conditions in both categories. Dyspepsia is common, and the subjective symptoms in either category varybetween patients. During a 3-month period, about 30% of the adult population suffers from dyspepsia [4]. Among these sufferers, only a minority has ulcer diseases (10%) [5] or reflux (12%) [6]. According to the literature, certain management strategies are recommended for some of these conditions, for example reflux [7]. For other conditions, for example functional dyspepsia, the evidence is more ambiguous [8-11]. One may expect a wider variation in the latter than in the former treatment strategies.

The aim of this study was to describe, using a qualitative approach, the variation in the management strategies and treatments suggested by clinicians in three different disciplines for patients with symptoms from the upper gastrointestinal tract. According to this aim, the hypotheses that guided our design were:

1. Management strategies and treatments suggested by different clinicians vary considerably, even when optimal treatment is clear cut, as documented by evidence in the literature.

2. Clinicians believe that the management strategies of their colleagues will be similar to their own.

## Methods

### Informants

Informants were selected to represent clinicians who regularly encountered patients with dyspeptic symptoms in their daily practice and who thus were expected to have a treatment policy for these patients. We therefore invited Swedish clinicians who were specialists in medical gastroenterology, surgery, and general practice to participate in this study. All clinicians gave their consent to be interviewed. They also suggested other clinicians who might be interested in participating in the study. Altogether, 27 clinicians participated, nine from each of the three specialities. This number is the number commonly used in studies that use judgement analysis to ensure a variation in answers [12].

The specialists in medical gastroenterology (four women and five men) all worked at Lund University Hospital at the time of the study. The surgeons (one woman and eight men) worked at Lund University Hospital or Helsingborg Hospital. The general practitioners (two women and seven men) worked in the Public Health Service in the southern part of Sweden. The clinicians were 35–56 years (average 47 years) of age and their experience in their speciality ranged from 1 to 22 years (average 10 years).

### Interviews and case scenarios

A useful approach for studying clinicians' strategies is judgement analysis [14], which describes the cognitive process involved in making a decision. It focuses on the actual decision made by the clinician and on which information, for example symptoms and clinical findings, that the clinician uses to reach that decision.

In our study, each of the clinicians was interviewed separately. The interviews were based on four case histories. The histories described frequent symptoms and findings in 30-year-old men seen in general practice and in specialist out-patient clinics (Table 1). Besides age and sex, all histories contained information on the localisation of symptoms, but information on subjective symptoms and findings from the examination varied from history to history. The questions, listed in Table 1, focused on how the clinician viewed whether there was a need for treatment, what management the clinician would suggest, which factors the clinician thought were most important to consider, and what decisions the clinician thought colleagues in their own speciality or in other specialities would make. The same questions were asked for each patient. The case histories were presented in the sequence described in Table 1. Thus, the questions were answered four times by each informant.

One of the authors (KK) with experience in judgement and decision analysis interviewed each informant at her or his office. Each interview began with the interviewer reading the first history. The interviewer then asked the questions aloud and wrote down the informant's answers after each question. If the informant wished, the history and questions were reread. At the end of the question-and-answer session after each case history, the interviewer read aloud the participant's answers and in cases of ambiguity, adjusted the answers. The interviews thus took the form of an oral questionnaire, but with the possibility to explain the answers more fully.

### Content analysis

The analysis was performed after all data had been collected from the informants. Two of the authors (KK, MT) with experience in qualitative studies read the interview notes. In a first step, each of the clinicians' answers was scrutinised to find content or evaluations in common, which were then coded as concepts. The following is an example of answers coded as a concept: To question A ("Do you think that it is a problem to decide whether there is a need for treatment in a case like this?" and if yes: "What problems?") informants gave answers like "there is no demonstrable disease, but the patient has symptoms" and "there is no diagnosis but a suffering patient" (patients 1 and 2). These answers were coded as the concept "The patient suffers, but there is no distinct diagnosis".

In the second step, related concepts were combined into categories, common to all the case histories. The authors compared their coding and resolved any differences through discussion to reach a consensus. The concepts and categories were compared with the original answers to ensure that the answers were covered by the classifications.

## Results

The varied answers of the clinicians are presented in Table 2, 3, 4. Different categories of problems existed that could explain the variation in the decision-making process. When answering the question "Do you think there is a problem to decide whether there is a need for treatment in a case like this?", five different categories of answers were given. For example, the basis for deciding whether or not to treat and what would be optimal treatment was ambiguous and, further, the evidence for treatment was poor. Clinicians also felt that they lacked competence in evaluating test results when making treatment decisions and that patient expectations and requirements were problems with patients 1–3. In these cases, the patients sometimes requested investigations and treatment, even though the clinician informed them that there was no reported evidence of effect in the literature for the requested treatment or investigation.

For the same patient, the management strategies considered by the different clinicians varied widely, including extensive examinations, non-pharmacological treatment, drug treatment, and surgery (Table 3). For patients 2 and 3, who had tested positive for Helicobacter pylori (H. pylori), some clinicians stated that they would suggest eradication treatment only if the patient had an ulcer, whereas other clinicians said they would suggest this treatment irrespective of the presence of ulcers. For patient 4, who had a duodenal ulcer, some clinicians suggested eradication treatment only if the patient tested positive for H. pylori, whereas others recommended that treatment irrespective of such test results. This variation was also observed among clinicians in the same speciality. Variation was more pronounced if the case history noted symptoms but no organic findings, as in patients 1 and 2, than if the case history noted unambiguous findings and symptoms, as in patients 3 and 4.

When asked "Which factors are most important to consider in your decision?" (Table 4), the clinicians defined three categories of information. For patients 1 and 2, whose symptoms were diffuse, several clinicians answered that the medical history was the most important piece of information to consider. They also claimed to need a more comprehensive medical history for patients 1 and 2 than for patients 3 and 4, whose symptoms were more obviously related to an organic diagnosis.

A majority of the clinicians believed that their treatment decisions would be similar to those of most of their colleagues in their speciality. Some gastroenterologists believed that surgeons would prefer surgery to medication, given the same test results. Several gastroenterologists and surgeons believed that family clinicians investigated less, informed more, and prescribed acid inhibitors on wider indications than gastroenterologists and surgeons.

## Discussion

### Methodological and results approaches

The four case histories in this study are representative of patients with conditions frequently seen in medical practice. Diseases of the gastrointestinal tract account for about 6% of all reported consultations in Sweden [13]. Gastritis, dyspepsia, and unspecified diseases in the ventricle and duodenum (patients 1 and 2) are diagnosed in 1.6% of the population; oesophagitis and reflux (patient 3) in 0.9%; and different ulcers (patient 4) in 0.6%.

The clinicians who participated in this study were not representative of a randomised selection of clinicians in Sweden but rather of a select group with a special interest in patients with symptoms from the gastrointestinal tract. Since their experience with these patients was extensive, they should have been able to develop a treatment policy. One would expect a variation in treatment strategy in this group to be more limited than among a randomised selection of clinicians.

The results underpin existing evidence that variations in medical practice exist [14-16]. This study presents variations both in treatment strategy and in what information is considered important for making treatment decisions. The histories of patients with diffuse symptoms but no objective findings (patients 1 and 2) gave rise to more extensive variations than did the case histories of patients 3 and 4 where the symptoms were more obviously related to a diagnosis (patient 3) or where more obvious organic changes existed (patient 4). Uncertainty in diagnosis could lead to uncertainty regarding which outcome of alternative interventions is optimal [17,18]. A meta-analysis that evaluates the most effective treatment in patients with functional dyspepsia [10] recommended the eradication of H. pylori if the treatment is to be effective from the patient's perspective, whereas other randomised, double-blind, controlled studies find that eradication has no beneficial effect [11]. These results were underpinned by the variations observed in this study.

Uncertainty due to lack of knowledge or professional competence may lead to qualitative differences. In this study, different management strategies were recommended for patients with reflux disease (patient 3), for example, eradication treatment as one strategy and changes in life-style as another. However, evidence in the literature presenting eradication as an optimal management of reflux is lacking [17]. Instead, this management strategy raises the cost to society and to the patient and causes unnecessary antibiotic pressure. Randomised, double-blinded, prospective studies conclude that the only indication for treating dyspepsia with proton pump inhibitors (PPIs) is the presence of an ulcer or reflux [7]. However, the majority of clinicians prescribed PPIs and H_2_-receptor antagonists for the treatment of dyspepsia, although no evidence of an effect with this treatment compared to placebo has been documented [9,10]. Thus, this regimen has led to high costs for society without any benefits. The drug costs for treatment of ventricular and duodenal ulcers and of reflux, including eradication treatment, were SEK 1.6 billion [USD 210 million] in 1998 in Sweden [13], while The Swedish Council on Technology Assessment in Health Care reports that society's direct and indirect costs for dyspepsia were between SEK 3.7 and 4.4 billion [USD 490 and 590 million] in 2000 [3].

From the informants' answers to the question "Which factors are most important to consider in your decision?", the completeness of some of the answers given in the interviews could be questioned. Most likely, the clinicians failed to answer the question in full as they left out many factors worthy of consideration. Other factors in the patient's life such as stress, mental mood, working situation, and diet were not mentioned by the clinicians in the interviews but should often be considered in treatment strategies in an actual clinical situation.

Within each of the three specialities, clinicians believed that their colleagues would treat patients in the same way as they themselves did. This aspect of "professional certainty" implies that clinicians believe that their practice is correct, irrespective of how much it in fact differs from that of other clinicians [18,19]. This unawareness of variation in the management of frequently seen patients may indicate a lack of communication and discussion about everyday cases. Such discussions are perhaps reserved for more "complex" and rare cases.

### Opportunities to change clinicians' practice

Among the causes of variation in medical practice, the influence of factors like patient characteristics (e.g. age, sex, morbidity, and personal preferences) could be more or less regarded as legitimate to explain variations in practice [20]. Other factors, like resource capacity, could be influenced by, for example, budget restrictions while management policy and practice style are more resistant to change [20]. These latter factors should therefore be the targets of efforts to change. However, many attempts to implement evidence by information alone, for example in the form of clinical practice guidelines, have failed to change management strategies [20-23]. Instead, a combination of methods is most likely needed if a permanent change is to occur [23]. Furthermore, it is of utmost importance that potential barriers are identified and that the clinicians who will be affected support the clinical practice guidelines to be implemented.

In recent years, the individual autonomy of patients and letting patients' preferences influence the choice of intervention have been emphasised [24]. In this study, such a strategy could explain why some clinicians mentioned that they felt pressured by patients to perform an intervention, even when the optimal strategy was non-intervention.

## Conclusion

In conclusion, our study adds to the present scientific literature by showing that, even in cases with a consensus in the scientific literature on treatment, clinicians can differ in their opinion of which management is optimal. Despite these variations, the clinicians believed that the decisions made by their colleagues would be similar to their own.

Overall, we as researchers must make scientific evidence comprehensible and communicate evidence in such a way that clinicians are easily able to interpret and implement it in practice. Of particular significance is that scientific evidence leads to an evidence-based care, which is effective clinical practice, and to the promotion of health from the perspective of the patient, together with cost-effectiveness as a priority. Cost-effectiveness is a vital concern. Targeting high patient satisfaction may lead to ineffectiveness if the economic consequences of a treatment strategy are ignored.

## Declaration of competing interests

The authors declare that they have no competing interests.

## Authors' contributions

Kerstin Knutsson, DDS, Odont dr, has special competence in judgement and decision-making within health care. KK contributed to the overall concept and design of the study and conducted the interviews. Grants from the Swedish Research Council (grant no. 521-2001-6341) supported this study.

Bodil Ohlsson, MD, PhD, is a specialist in gastroenterology and contributed with her knowledge in this area to the design of the study and the interpretation of the data.

Margareta Troein, MD, PhD, has special competence in qualitative methods in medical care and contributed to the qualitative analysis and interpretation of the data.

All authors read and approved the final manuscript.

## Pre-publication history

The pre-publication history for this paper can be accessed here:



## Figures and Tables

**Table 1:** Case histories and questions presented to the informants.

Patient 1. A 30-year-old man with symptoms from the upper part of the abdomen. The patient has had intermittent pain, made worse by stress, for a few years. One year ago an ultrasound examination and an oesophagogastroduodenoscopy were performed. No abnormal findings were registered. H. pylori serology was negative.
Patient 2. A 30-year-old man with symptoms from the upper part of the abdomen. The patient has had intermittent pain, made worse by stress, for a few years. One year ago an ultrasound examination and an oesophagogastroduodenoscopy were performed. No abnormal findings were registered. H. pylori serology was positive.
Patient 3. A 30-year-old overweight man with symptoms from the upper part of the abdomen and retrosternally. The pain worsens when he leans forward and when he rests in a prone position. He sometimes experiences a sour or bitter taste in his mouth. H. pylori serology was positive.
Patient 4. A 30-year-old man with symptoms from the upper part of the abdomen. The ultrasound examination revealed nothing abnormal, but the oesophagogastroduodenoscopy indicated a duodenal ulcer.
After each case the following questions were asked: A. Do you think that it is a problem to decide whether there is a need for treatment in a case like this? If yes: What problems? B. How would you manage a case like this? C. Which factors do you think are most important to consider in your decision? D. What do you believe your colleagues would decide in a case like this?

**Table 2:** Clinicians' answers coded as concepts and organised into categories. Answers of 27 clinicians to the question "Do you think that it is a problem to decide whether there is a need for treatment in a case like this?" If the physicians admitted that it was a problem, they were then asked "What problems?" Answers were coded as concepts and organised into categories for each patient. Numbers of answers are presented within parentheses. Each clinician may give several answers that could be included in different categories.

*Categories*	*Concepts*			
	
	*Patient 1*	*Patient 2*	*Patient 3*	*Patient 4*
There is no problem to decide	(16)	(20)	(19)	(22)
Basis for treatment decision is insufficient	There is no diagnosis (5)	There is no diagnosis (3)	The amount of discomfort is not clear (1) More examinations are needed (2)	No examination of Helicobacter pylori has been made (3)
Evidence for treatment is poor	There is hardly any evidence for benefits of treatment (1) It is difficult to treat dyspepsia (1) Patient suffers, but there is no distinct diagnosis (2) Psychosomatic problems (2)	Patient suffers, but there is no distinct diagnosis (1)	Recurrent condition (2)	
Clinician is uncertain of her or his own competence		Uncertainty about indication for treatment when Helicobacter pylori serology is positive (1)	Uncertainty about indication for treatment when Helicobacter pylori serology is positive (1)	
Patient's expectations	Patient wishes treatment (1)	Patient requires treatment due to positive Helicobacter pylori serology (2)	Patient is not motivated to reduce weight (1) Patient requires treatment due to positive Helicobacter pylori serology (2)	

**Table 3:** Clinicians' answers coded as concepts and organised into categories. Answers of 27 clinicians to the question "How would you manage a case like this? " Answers were coded as concepts and organised into categories for each patient. Numbers of answers are presented within parentheses. Each clinician may give several answers that could be included in different categories.

*Categories*	*Concepts*			
	
	*Patient 1*	*Patient 2*	*Patient 3*	*Patient 4*
Extend the examination	Exclude coeliac disease, examine colon, liver, gall bladder (6) Evaluate patient's psychosocial conditions (4) Evaluate outcome of previous treatment (3)	Oesophagogastroduodenoscopy, expiration tests for Helicobacter pylori (10) Evaluate patient's psychosocial condition (2) Evaluate outcome of previous treatment (1)	Oesophagogastroduodenoscopy, expiration tests for Helicobacter pylori (18)	Diagnostic tests for Helicobacter pylori (8)
Prescribe non-pharmacological treatment	Reassuring information (8) Expectancy (3) Dietary advice, reduction of alcohol intake (11) Reduction of stress (7)	Reassuring information (7) Expectancy (4) Dietary advice (6) Reduction of stress (3)	Smoking cessation (2) Weight reduction (3) Dietary advice (3) Unspecific life-style changes (6)	Unspecific life-style changes (1)
Prescribe drugs against acidity	Antacids (7) H2-receptor antagonists (13) Proton pump inhibitors (5) Sucralphate (1)	Antacids (7) H2-receptor antagonists (9) Proton pump inhibitors (6) Sucralphate (1)	Antacids (1) H2-receptor antagonists (8) Proton pump inhibitors (15)	Proton pump inhibitors (11)
Prescribe triple treatment (antacid and antibiotics against Helicobacter pylori)		Only if patient has an ulcer (7) Yes, irrespective of whether patient has an ulcer (4)	Only if patient has oesophagitis or an ulcer (1) Yes, irrespective of whether patient has oesophagitis or an ulcer (1)	Only if patient has a positive test for Helicobacter pylori (11) Yes, irrespective of whether Helicobacter pylori serology is positive (14)
Prescribe other drugs	Drugs which increase gut motility (2) NSAID (1) Bulking agent (1)	Drugs which increase gut motility (1)	Alginate (6)	
Recommend surgery			If patient has hiatus hernia (1)	

**Table 4:** Clinicians' answers coded as concepts and organised into categories. Answers of 27 clinicians to the question "Which factors are most important to consider in your decision?" Answers were coded as concepts and organised into categories for each patient. Numbers of answers are presented within parentheses. Each clinician may give several answers that could be included in different categories.

*Categories*	*Concepts*			
	
	*Patient 1*	*Patient 2*	*Patient 3*	*Patient 4*
Medical history	Patient's age (4) Symptoms including localisation of pain (12) Life-style/stress (12) Effects of previous medication (1) Similar problems for a long time (3)	Patient's age (5) Symptoms including relation to meals (12) Effects of previous medication (1) Similar problems for a long time (2)	Patient's age (2) Symptoms generally and in relation to position (21)	General state of health (1) Symptoms (1)
Results from examinations	Oesophagogastroduodenoscopy and ultrasound (6) Test for Helicobacter pylori (5)	Oesophagogastroduodenoscopy and ultrasound (1) Test for Helicobacter pylori (7)	Oesophagogastroduodenoscopy (3) Test for Helicobacter pylori (1) Weight (1)	Oesophagogastroduodenoscopy (26) Test for Helicobacter pylori (3)
Patient's expectations	Patient's preferences (1)	Patient requires treatment due to positive Helicobacter pylori serology (3)	Patient requires treatment due to positive Helicobacter pylori serology (3)	
